# New Insights into *N*-Doped Porous Carbons as Both Heterogeneous Catalysts and Catalyst Supports: Opportunities for the Catalytic Synthesis of Valuable Compounds

**DOI:** 10.3390/nano13132013

**Published:** 2023-07-05

**Authors:** Elena Pérez Mayoral, Marina Godino Ojer, Márcia Ventura, Ines Matos

**Affiliations:** 1 Departamento de Química Inorgánica y Química Técnica, Facultad de Ciencias, Universidad Nacional de Educación a Distancia (UNED), Urbanización Monte Rozas, Avda. Esparta s/n Ctra. de Las Rozas al Escorial Km 5, Las Rozas, 28232 Madrid, Spain; 2 Facultad de Ciencias Experimentales, Universidad Francisco de Vitoria (UFV), Ctra. Pozuelo-Majadahonda Km 1.800, Pozuelo de Alarcón, 28223 Madrid, Spain; marina.godino@ufv.es; 3 LAQV/REQUIMTE, Departamento de Química, Faculdade de Ciências e Tecnologia, Universidade Nova de Lisboa, 2829-516 Caparica, Portugal; mm.ventura@fct.unl.pt

**Keywords:** *N*-doped porous carbons, nanomaterials, green chemistry, fine chemical synthesis

## Abstract

Among the vast class of porous carbon materials, *N*-doped porous carbons have emerged as promising materials in catalysis due to their unique properties. The introduction of nitrogen into the carbonaceous matrix can lead to the creation of new sites on the carbon surface, often associated with pyridinic or pyrrolic nitrogen functionalities, which can facilitate various catalytic reactions with increased selectivity. Furthermore, the presence of N dopants exerts a significant influence on the properties of the supported metal or metal oxide nanoparticles, including the metal dispersion, interactions between the metal and support, and stability of the metal nanoparticles. These effects play a crucial role in enhancing the catalytic performance of the *N*-doped carbon-supported catalysts. Thus, *N*-doped carbons and metals supported on *N*-doped carbons have been revealed to be interesting heterogeneous catalysts for relevant synthesis processes of valuable compounds. This review presents a concise overview of various methods employed to produce *N*-doped porous carbons with distinct structures, starting from diverse precursors, and showcases their potential in various catalytic processes, particularly in fine chemical synthesis.

## 1. Introduction

Carbon-based materials, as an enormous family of functional materials, currently present a huge and increasing number of ecological applications in different domains [1,2]. These materials have been specifically considered for a long time almost as ideal catalysts or catalysts supports, with recognized efficiency in fine chemical synthesis. The combination of their porous structure and chemical surface provides the appropriate chemical environments to favor important chemical transformations. Different functionalities can be present on the carbon surface, including oxygenated functions or doping with foreign species, mainly depending on the used sources, with the carbonization and activation processes focusing on enhancing the catalytic performance [3]. 

Nitrogen-doped carbons have attracted much attention in the development of environmentally advanced functional materials. Among the most recent applications, we highlight CO_2_ capture and energy conversion and storage [4] but also these materials acting as supports of metals [5,6] or even metal-free catalysts [7]. Compared with undoped porous carbons, the *N*-doped carbons are clearly the preferred supports for both metal and metal oxides. *N*-doping increases the creation of basic sites, offering benefits in several organic transformations such as hydrogenation and dehydrogenation, oxidation, and coupling reactions, among others [8]. Note that *N*-doping also modifies the catalyst’s properties; for example, it notably increases the wettability, which favors the catalyst’s dispersion and stability through stronger metal–support interactions. Therefore, metals supported on *N*-doped carbon catalysts emerge as a future opportunity in heterogeneous catalysis research.

In fact, much more recently, these materials have been being investigated with the excellent possibility of designing single-atom catalysts (SACs), in which transition metal species—mainly Co, Cu, Zn, Pd, and Ni, among others—are stabilized on the carbon surface and applied in organic synthesis [9]. The presence of single metal atoms homogeneously distributed and anchored onto a support facilitates specific interactions with reactants, often enhancing the catalytic performance. SACs combine the advantages of both homogeneous and heterogeneous catalysts, providing highly selective isolated active sites. In general, due to the thermal treatments necessary for the preparation of solid catalysts, although also during their application, metal aggregation can be produced. For this reason, the control of the coordination environments on the support to stablish strong coordination bonding with metal atoms is of capital importance and a challenge in the development of SACs [10]. In this context, metal–organic frameworks (MOFs) are recognized as ideal platforms to develop SACs with well-defined structures and tunable porosity levels [11], particularly *N*-doped porous carbons from zeolitic imidazolate frameworks (ZIFs) [12]. 

**Figure 1:**
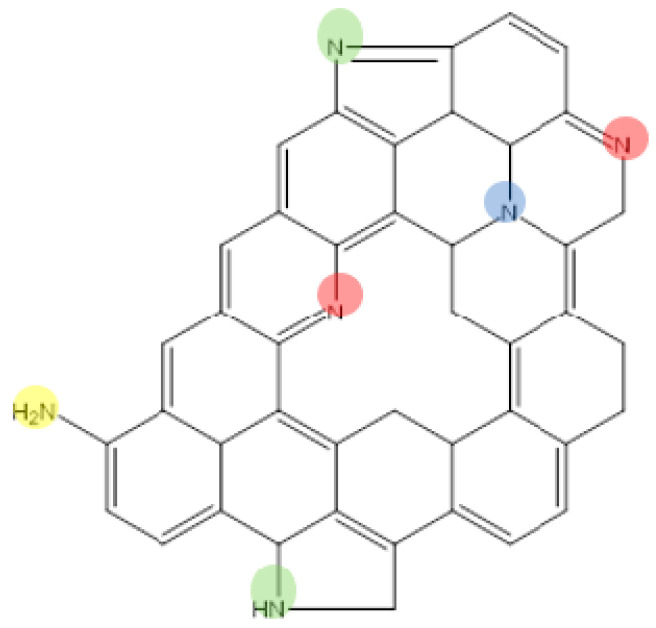
Schematic representation of the nitrogen groups that can be present on the carbon material’s surface. Pyrrolic (*green*); pyridinic (*red*); graphitic (*blue*); amine (*yellow*).

On the surface of the N-doped carbons, three types of nitrogen (N) atoms can be identified, namely pyridinic, pyrrolic, and graphitic [13]. These surface configurations are represented in Figure 1. Pyridinic-N is bonded to two carbon (C) atoms, forming a a hexagon, while pyrrolic-N is bonded to two C atoms, forming a pentagon. Both types of nitrogen can occur at the edges of the graphene layer but also within the layer, often in association with vacancies. The graphitic N replaces a carbon atom in the graphene layer and forms bonds with three neighboring C atoms in an *sp*^2^ configuration [14]. For graphitic N, two configurations can be expected also, one near the layer edge and the other inside the carbon layer. Additionally, primary and secondary nitrogen functional groups with hydrogen bonds can be present [15].

To establish the specific influence of the different types of N configurations over the properties is not an easy task, since it is not easy to control what kind of configuration will be obtained; as a consequence, the relationships between the structures and properties remain an active field of research to further explain the observed effects of doping [15,16,17]. 

This review briefly covers the different methods used to produce *N*-doped porous carbons from structurally different precursors—including biomass and waste products, distinct carbon forms, and other emerging precursors, such as MOFs and COFs—as well as the main characteristics, although mainly focusing on their application to produce highly valuable compounds. Although an extensive family of *N*-doped carbon catalysts is summarized, including metal-free and metal-supported catalysts, comprising traditional but also more advanced materials, this paper seeks to show the new horizon and probably the future trends in the green synthesis of fine chemicals. It is important to note that *N*-doped carbons have presented an opportunity to work up the heterogeneous basic catalysis method based on fine chemical synthesis, which is notably less developed than the acid method.

## 2. *N*-Doped Porous Carbons: Precursors and Synthesis Strategies

The production of *N*-doped porous carbons has been reported using several different methods and strategies. The most common include the carbonization of precursors with high nitrogen contents and the post-synthesis modification of the carbon surface via the grafting or impregnation of nitrogen-containing groups. *N*-doping introduces heteroatoms into the carbon matrix, altering its electronic and surface properties and increasing its functionality. The choice of the precursor and synthesis method depends on the desired properties and applications of the final material. These materials are of particular interest in areas of electrochemical applications such as electrodes for supercapacitors and other energy-related topics but also in environmental remediation. During catalysis, the presence of nitrogen atoms in the carbon material is of great interest either to introduce new active functional groups or to tailor the support for metal ions in the material [18,19,20].

Thus, *N*-doped carbon catalysts have emerged as a promising class of heterogeneous catalysts for various organic transformations, including C-C and C-X bond formation reactions, such as Knoevenagel condensation, aldol reaction, and Suzuki coupling, as well as electrocatalytic processes such as the hydrogen evolution reaction (HER), oxygen evolution reaction (OER), and oxygen reduction reaction (ORR) [21].

It is worth noting that the synthesis and optimization of *N*-doped carbon catalysts for specific chemical synthesis applications involve various parameters, such as the nitrogen precursor, carbonization temperature, and specific nitrogen functional groups that are desired. Moreover, the incorporation of additional heteroatoms (such as phosphorus, sulfur, or boron) in conjunction with nitrogen can further enhance the catalytic performance of *N*-doped carbon materials [22,23].

### 2.1. Biomass- and Waste-Derived N-Doped Carbons

The carbon source is a critical component in the synthesis of *N*-doped porous carbons. One of the commonly used precursors for porous carbons and *N*-doped porous carbons is biomass [24], which includes natural products such as lignin [25], cellulose [26], and chitin [27] or other plant-based materials [28]. Biomass is a renewable and sustainable source of carbon, and its utilization can reduce dependence on fossil fuels. The carbonization of biomass in the presence of nitrogen-containing compounds or a nitrogen source, such as ammonia or melamine, can lead to the formation of *N*-doped porous carbons [29].

**Figure 2:**
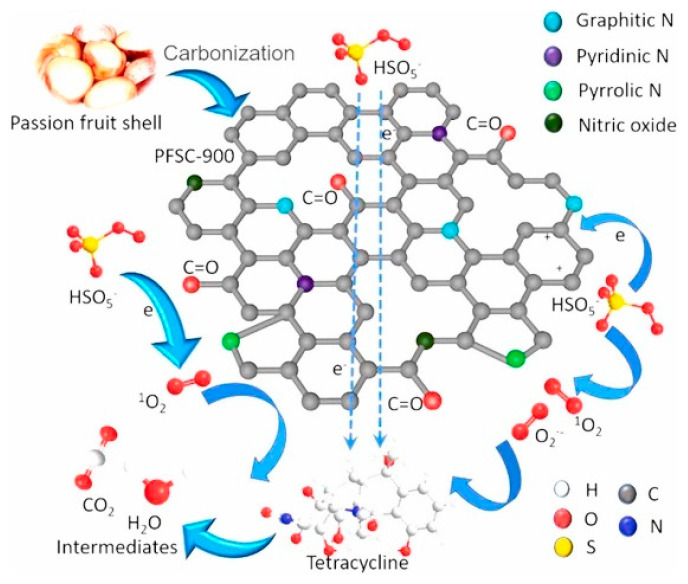
Graphical representation of the carbon surface obtained from passionfruit shells, including a representation of the types of *N*-containing functional groups on the carbon surface. Copyright © 2021, Elsevier B.V. [37].

The carbonization process involves heating the biomass a to high temperature (e.g., 800 °C) under controlled conditions in the absence of oxygen, resulting in the decomposition of the organic components and the formation of carbon-rich materials. During the carbonization, nitrogen-containing compounds or a nitrogen source (e.g., ammonia, melamine) can be introduced to incorporate nitrogen into the carbon structure, see Figure 2 [30,31,32]. The nitrogen sources react with the carbon precursors leading to the formation of *N*-doped carbons with tailored nitrogen functionalities [20].

The single-step co-hydrothermal treatment of biomass with a nitrogen source such as urea is another reported method for successfully enriching carbon structures [33].

The post synthesis method of incorporating nitrogen atoms involves the treatment of pristine biochar with the *N*-containing precursor. The ammonification of the carbon with ammonia is a commonly reported method [34]. The previous oxidation of the carbon surface has been reported to favor an increase in the N content in the final carbon [35]. Other nitrogen sources have been reported, such as urea [36].

Another sustainable carbon source includes waste materials, such as food waste, sewage sludge, municipal solid waste, industrial waste, or waste plastics, which can also serve as precursors for *N*-doped carbons. These waste materials are often abundant and readily available, making them attractive candidates for sustainable carbon synthesis. The waste materials can undergo a similar carbonization process as biomass followed by nitrogen doping to obtain *N*-doped carbons. The incorporation of nitrogen during carbonization can be achieved by introducing nitrogen-rich compounds or waste materials that already contain nitrogen functionalities. Waste-derived *N*-doped carbons have the potential for not only reduced waste disposal but also to produce valuable carbon materials that can be used in various applications, while also addressing waste management challenges [38].

In both cases, the synthesis of *N*-doped carbons from biomass or waste typically involves additional steps to enhance the porosity and surface area of the resulting materials. Activation processes, such as chemical or physical activation, can be employed to create a porous structure in the carbon material. Chemical activation involves the treatment of the carbonized precursor with an activating agent, such as potassium hydroxide (KOH) [39] or phosphoric acid (H_3_PO_4_) [40], which creates pores and increases the surface area. Many other activating agents are reported in the literature, including potassium carbonate (K_2_CO_3_) [41] and zinc chloride (ZnCl_2_) [42], along with bio-derived ones such as phytic acid [43]. Physical activation, on the other hand, utilizes steam or carbon dioxide activation to generate porosity [36,44].

One of the major advantages of biomass- and waste-derived *N*-doped carbons is their low cost compared to traditional carbon materials. These materials can also be easily synthesized in large quantities, making them suitable for commercial applications. Their low cost, sustainability, and unique properties make them an attractive alternative to traditional carbon materials, and their production from waste materials can also contribute to environmental sustainability [45].

### 2.2. Covalent Organic Frameworks (COFs) and Metal–Organic Frameworks (MOFs) as Precursors for N-Doped Carbons

Covalent organic frameworks (COFs) and metal–organic frameworks (MOFs) are highly ordered and have well-defined and tunable structures, thereby offering an attractive starting point for the synthesis of *N*-doped carbons [46]. 

COFs are composed of organic building blocks linked by covalent bonds, creating extended crystalline structures. These frameworks can be designed with specific pore sizes, surface areas, and functionalities by selecting suitable building blocks [1].

In COFs, the nitrogen atoms are typically introduced by using a nitrogen-containing building block during the synthesis process. A common approach involves the use of a triazine-based building block to introduce nitrogen atoms into the COF structure. After the COF is synthesized, it can be explored as a precursor for *N*-doped carbons by subjecting them to high-temperature pyrolysis or chemical activation processes in the presence or not of nitrogen-rich precursors. During these processes, the organic components decompose, leaving behind a carbon framework with incorporated nitrogen atoms derived from the precursor. The resulting *N*-doped carbons exhibit enhanced catalytic activity, electrical conductivity, and stability compared to their pristine carbon counterparts [47,48,49].

On the other hand, MOFs are constructed from metal ions or clusters connected by organic ligands. MOFs offer exceptional structural diversity and porosity, making them promising precursors for the synthesis of *N*-doped porous carbons by incorporating nitrogen-containing ligands into the MOF structure. The strategy for preparing *N*-doped carbons from MOFs involves the carbonization of the organic ligands while retaining the metal component in a reduced or metallic form. This process can also be achieved using thermal treatment or chemical activation methods. The resulting *N*-doped carbons possess a hierarchical pore structure, large surface area, and improved nitrogen content, and often highly dispersed metal sites, which contributes to their enhanced performance in various applications [50,51,52].

### 2.3. Carbon Nanotubes, Nanofibers, and Graphene N-Doped Materials

Carbon nanotubes (CNTs) are cylindrical structures composed of carbon atoms arranged in a hexagonal lattice, which may be single-walled carbon nanotubes (SWCNTs) or multiwalled carbon nanotubes (MWCNTs), depending on the number of graphene sheets that compose their walls. Meanwhile, graphene is a single layer of carbon atoms arranged in a two-dimensional hexagonal lattice. These highly structured carbon materials present extremely interesting properties and have found applications in many distinct fields. Both materials can be doped with nitrogen to introduce new properties and enhance their performance [53].

*N*-doping can be achieved by introducing nitrogen atoms into the carbon lattice, either during the synthesis process or through a post-synthesis treatment [54,55]. The incorporation of N heteroatoms during the production process includes high-temperature arc discharge [56,57], chemical vapor deposition (CVD) [58], and solvothermal procedures [59]. 

The post-synthesis treatments may include the grafting of N-containing molecules [60], encapsulation of compounds of interest [61], or polymer wrapping [62].

**Figure 3:**
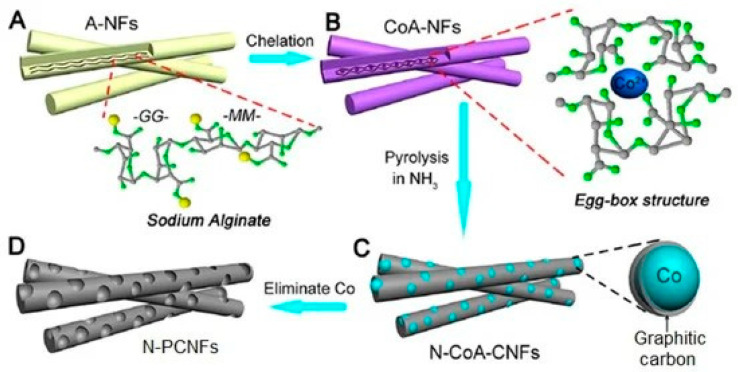
Schematic illustration of the synthesis process of N-PCNFs: (**A**) spinning materials; (**B**) cobalt alginate nanofibers (CoA-NFs); (**C**) Co NP decorated N-doped carbon nanofibers; (**D**) mesoporous N-doped carbon nanofibers [64]. Copyright © 2015, American Chemical Society (further permission related to the material excerpted should be directed to the ACS).

Dai et al. [63] first reported the development and application of carbon nanotubes doped with nitrogen used an ORR. Since then, many reports can be found in the literature, including the electrospinning of biomass-derived precursors into carbon nanofibers (CNF), an example can be found in Figure 3 [64,65].

### 2.4. Carbonization of Nitrogen-Containing Synthetic Materials

Other precursor for *N*-doped carbons include synthetic materials containing nitrogen in their structure. These can be either waste materials such as plastic-derived waste or synthesized materials tailored for this purpose. Polyacrylonitrile, polyamide, polyimide, polyvinylpyridine, polyaniline, acetonitrile, polypyrrole, melamine–formaldehyde, and urea–formaldehyde resins are some examples [66,67,68,69,70].

Of particular interest have been the formaldehyde gels or foams [71,72]. Formaldehyde (HCHO) is a small molecule containing carbon, hydrogen, and oxygen, and it can readily undergo polymerization reactions to form more complex carbon-based structures. When formaldehyde is combined with appropriate nitrogen sources, such as ammonia or *N*-containing organic compounds such as urea or melamine, nitrogen atoms can be incorporated into the carbon matrix during the synthesis process [73]. 

Another interesting and emerging precursor group contains ionic liquids (ILs) [74], due to their negligible vapor pressure and high thermal stability. However, their use as carbon precursors is very dependent on the anion and cation structure, whereby ILs containing cross-linkable moieties seem to be more favorable for direct carbonization [75]. In this context, (poly)ionic liquids (PILs) have also been used as carbon precursors [76]. 

An alternative method is the mixture of ILs with additional carbon sources [74], such as biomass-derived molecules or residues [77]. ILs can also be used as nitrogen sources in the production of *N*-doped materials, as reported by She et al. [78] for the *N*-doping of graphene.

For enhanced control of the porosity, the hard template method is used, where nanoparticles (such as silica) are used as a sacrificial template to be removed later in the synthesis processes, increasing the porous space [79]. This method can be used with ILs but also with many other polymeric precursors. It is important to mention that the already presented physical and chemical activation steps for enhance porosity can also be applied to the carbons obtained from the precursors summarized in this section.

### 2.5. Metal-Supported N-Doped Porous Carbons

Metal-supported *N*-doped porous carbons are composite materials where metal nanoparticles or catalysts are anchored onto a porous carbon matrix that has been previously modified or doped with nitrogen. Whereas the *N*-doping enhances the surface properties of the carbon material, such as its adsorption capacity and catalytic activity, making it suitable for various applications in catalysis, energy storage, and environmental remediation, the metal nanoparticles supported on *N*-doped porous carbons act as active sites for chemical reactions. This combination, along with the high surface stability level provided by the porous carbon, creates a synergistic effect, leading to improved performance and efficiency in various chemical processes. 

Metal-supported *N*-doped porous carbon composites have gained significant attention in the past decade as a promising material for heterogeneous catalytic reactions. These materials have found wide applications in the energy and environmental sectors. They have been utilized in various areas such as batteries [80,81,82,83,84,85,86], supercapacitors [87,88], and electrocatalysis [89,90,91,92].

The introduction of transition metals as active sites in *N*-doped carbon supports enhances the catalytic performance and enables broader applications. Metals can coordinate with nitrogen atoms, forming M-N_x_ groups (M = Fe, Co, Ni, Cu, Zn, etc.), and influence the catalyst’s electronic structure by modifying the local coordination environment. *N*-doping alters the physical and chemical properties of carbon materials, enhancing the interaction between the metal and support. This optimization facilitates charge transfer redistribution and enhances the adsorption energy on the substrate and the amount of intermediate adsorption the metal surface, ultimately improving the reaction performance [93,94]. These metal–support interactions impact the adsorption, nucleation, growth, and deposition of metal species during preparation, leading to changes in metal dispersion and crystallinity, which are crucial for their catalytic performance [95,96]. Wei et al. [97] demonstrated that noble metal nanoparticles of Pd, Pt, and Au can undergo transformation into thermally stable single atoms at temperatures exceeding 900 °C in an inert atmosphere. This conversion is driven by the formation of a more thermodynamically stable noble metal–*N* structure, as predicted by a noble metal–N4 structure model, which occurs when mobile noble metal atoms become captured by defects in *N*-doped carbon. Notably, the thermally stable Pd single atoms exhibit superior activity and selectivity compared to Pd nanoparticles in the semihydrogenation of acetylene. In a similar study, Ni metal nanoparticles supported on *N*-doped carbon with abundant defects underwent a transformation into thermally stable Ni single atoms [98]. This synthesis process not only converted the nanoparticles into single atoms but also generated numerous pores, which facilitated the interaction between dissolved CO_2_ and the single Ni sites, thereby enhancing the catalytic performance of the resulting composite material. The proposed mechanism suggested that the Ni nanoparticles can break surface C@C bonds and penetrate the carbon matrix, leaving surface pores. Upon exposure to *N*-doped carbon, the strong coordination forces separate Ni atoms from the Ni nanoparticles. The experimental results from CO_2_ electroreduction testing revealed that surfaces enriched with Ni single atoms delivered superior performance compared to supported Ni nanoparticles and other similar catalysts.

The dispersion of metals on a suitable *N*-doped carbon support is key for achieving enhanced catalytic performance when preparing metal-supported *N*-doped carbon composites for various catalytic applications. A higher dispersion rate leads to increased surface atoms, and consequently higher catalytic activity. Moreover, the choice of the metal precursors, carbon supports, and synthesis process significantly impacts the catalyst’s performance [99,100]. Therefore, selecting an appropriate synthesis method for metal-supported *N*-doped carbon composites should be based on the characteristics of the metal or metal oxide, as well as the *N*-doped carbon. There are several methods for the synthesis of metal–*N*-doped carbon composites, which can be grouped into two main categories: (i) the post-loading method, which involves depositing metal precursors onto pre-synthesized *N*-doped carbon materials using techniques such as impregnation and deposition–precipitation; (ii) the simultaneous introduction of metal precursors and N species on the pre-synthesized carbon materials [96,101,102]. The preparation of supported metal catalysts can be easily accomplished using post-loading methods such as impregnation and deposition precipitation when the *N*-doped carbon materials are available. CVD has also been applied as a post-loading method for the fabrication of these materials [103,104,105,106]. The immobilization process heavily depends on the surface chemistry of the support, with a crucial role played by the incorporated N atoms. These N atoms enhance the basicity and hydrophilicity of the carbon atoms, thereby facilitating the deposition of the metal atoms [102]. Normally, after the deposition step, a final reduction step is needed to reduce the metal on the deposited salt into the metallic state. Reducing agents such as H_2_, HCHO, N_2_H_4_·H_2_O, or NaBH_4_ can be used [96]. Impregnation is an efficient and easily scalable technique that is suitable for industrial production. Among the referred post-loading methods, impregnation is the most frequently used preparation method due to its simple execution and low waste streams [102]. In this method, a metal precursor, typically a salt such as a metal nitrate, acetate, chloride, sulfate, carbonate, or organic metal complex, is dissolved in an appropriate solvent. The porous *N*-doped carbon material is then added to the metal salt solution with stirring. After solvent removal, the resulting solid is subjected to oven-drying, calcination, or reduction before being tested as a catalyst. The deposition–precipitation method was originally developed to produce catalysts with higher metal loading levels compared to impregnation, which is limited by solubility. It involves the dissolution of the metal precursor followed by a change in pH, temperature, or evaporation to achieve the complete precipitation of metal compounds as metal hydroxides onto the surfaces of *N*-doped carbon materials. A homogeneous particle size distribution can be achieved under appropriate conditions; however, controlling the nucleation process of the metal species can be challenging, requiring careful management to prevent local concentrations from exceeding critical supersaturation. Moreover, an excess of reductant is necessary to ensure the complete formation of metal nanoparticles [102].

The second category, involving the simultaneous introduction of metal precursors and N species on the carbon materials, offers an improved and more efficient route for obtaining metal-supported *N*-doped carbon composites, circumventing the need for extensive post-loading procedures. A pyrolysis process can be employed to obtain metal catalysts supported on *N*-modified carbon materials. The use of organometallic amine complexes as precursors enables this process; during pyrolysis, the moiety complexes undergo partial or complete decomposition, resulting in the formation of active metal nanoparticles [107,108]. Alternatively, heating a mixture of metal salts, *N*-containing sources, and carbon at high temperatures (>600 °C) yields similar metal nanocatalysts supported on *N*-doped carbon [109]. In this approach, inexpensive and readily available inorganic metal salts such as acetates, sulfates, and chlorides can be utilized. Common N sources include melamine, 1,10-phenanthroline, dicyandiamide, tripyridyl, and triazine [102,110,111]. The choice of starting materials influences the microstructures of the catalysts obtained, leading to notable variations in catalytic performance. Usually, catalysts obtained through this method often exhibit a broad size distribution of nanoparticles. The heterogeneity of such catalysts complicates the identification of the true catalytically active sites, reduces their atomic efficiency, and may lead to undesirable side reactions. 

**Figure 4:**
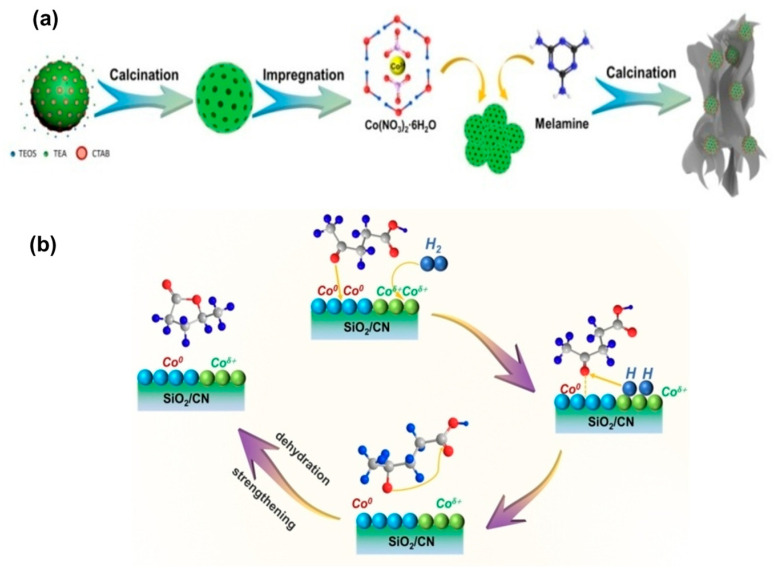
Schematic procedure for the preparation of the catalyst Co@mSiO_2_–CN3 (**a**) and a possible reaction mechanism for the conversion of levulinic acid to γ-valerolactone (**b**). Adapted from [112].

As an example of a synthesis strategy and the application of a metal-supported *N*-doped carbon catalyst, Zhang et al. [112] presented a simple impregnation mixing pyrolysis strategy to construct a highly efficient catalyst, Co@mSiO_2_–CN3, with improved metal dispersion and enhanced Lewis acidity, benefiting from the introduction of *N*-doped carbon materials, which could completely convert levulinic acid to γ-valerolactone under mild conditions. In Figure 4, we present the schematic procedure for the preparation of the catalyst (a) and a possible reaction mechanism for the conversion of levulinic acid to γ-valerolactone (b) [112].

Metal-supported *N*-doped carbons have shown promising catalytic applications in various fields. Some of the notable catalytic applications include the ORR and OER processes [113,114]. These catalysts help to facilitate the conversion of oxygen into water, playing a crucial role in energy conversion and storage technologies (ORR), and can enhance the kinetics of oxygen evolution, leading to improved efficiency and performance during water splitting and energy generation in processes such as electrolysis and in rechargeable metal–air batteries (OER) [115]. Metal-supported, *N*-doped carbons have also been explored as catalysts for the hydrogen evolution reaction (HER), which is an essential reaction in electrochemical hydrogen production. These catalysts can reduce the overpotential required for hydrogen evolution, making the process more efficient and sustainable [116]. Metal-supported, *N*-doped carbons have been also investigated as catalysts for carbon dioxide (CO_2_) reduction reactions, aiming to convert CO_2_ into valuable fuels or chemical feedstocks. These catalysts can facilitate the electrochemical or photochemical reduction of CO_2_, contributing to carbon capture and utilization strategies [117,118]. These catalysts can effectively degrade organic contaminants, decompose harmful pollutants, and contribute to sustainable remediation technologies. There are a few examples for the application of these materials for environmental remediation purposes, such as the removal of pollutants from water and air [119,120]. Finally, metal-supported, *N*-doped carbons have shown potential in various organic chemical reactions, including C-C bond formation [121,122], hydrogenation [95,123], oxidation [124,125,126], and coupling reactions [116,127]. The catalysts can exhibit high selectivity, activity, and stability, enabling efficient and environmentally friendly synthesis routes.

The utilization of metal supported on *N*-doped carbon catalysts holds great potential for advancing the heterogeneous catalysis process in the future. In summary, the incorporation of N dopants into carbon-supported metal or metal oxide catalysts presents clear advantages over their undoped counterparts in heterogeneous catalysis. These advantages encompass improved catalytic performance through enhanced basicity and wettability, as well as the positive influence on the properties of the supported metal or metal oxide nanoparticles.

## 3. *N*-Doped Porous Carbon Materials in Fine Chemicals Synthesis

The development of *N*-doped porous carbons is acquiring great importance, contributing to the search for new basic catalysts, which are notably less studied than the acidic ones, although not only because those mainly doped with transition metals are also investigated in chemoselective oxidation–reduction reactions. Note that base-catalyzed reactions are key in the preparation of products demanded by society such as drugs, fragrances, or even chemical intermediates. This section consists of an overview concerning the functional *N-*doped porous carbons as metal-free or transition-metal-doped catalysts applied in fine chemical synthesis.

### 3.1. Metal-Free N-Doped Porous Carbon Catalysts 

Metal-free porous carbon catalysts present an extraordinary importance from an environmental point of view [128]. Specifically, metal-free *N*-doped porous carbons have been principally investigated in acid–base and oxidation reactions, as summarized below.

#### 3.1.1. Base-Catalyzed Reactions

***Knoevenagel condensation*:** This condensation method is one of the most studied acid–base reactions, considered as a test reaction to determine the basicity depending on the reaction conditions and the number of active sites and texture of the catalysts. In the last decade, Dommele et al. [129] has reported on different *N*-containing carbon nanotubes (NCNTs) active for Knoevenagel condensation between benzaldehyde and ethylcyanoacetate. The NCNTs were obtained via the CVD of acetonitrile and pyridine as carbon and nitrogen precursors over Co or Ni supported on SiO_2_ catalysts at different temperatures. The basic functions are mainly attributed to the pyridinic and quaternary nitrogen species located at the edges of the graphene sheets or in defects and within the sheets, respectively. The amounts of both species can vary with the temperature used in the synthesis process in such a manner that pyridinic nitrogen is formed under low temperatures (550 °C), whereas quaternary nitrogen is formed at higher temperatures (750 °C). The catalytic activity level (ranging 2–8·10^−3^ mol product h^−1^ g^−1^ catalyst) seems to be related to the pyridinic nitrogen at the accessible catalyst surface and is comparable to those reported for other basic catalysts such as hydroxyappatites and aluminophosphate oxynitrides. Similarly, Wang et al. [130] described a series of NCNTs also prepared via CVD but using the catalyst Fe-SBA-15, showing high nitrogen loading (16.6 mmol g^−1^; more than the NCNTs mentioned above at 7.4 mmol g^−1^) [129]. The precursors used—ethylene diamine, diethylamine, or dimethylamine—influenced both the yield and N/C atomic ratio of the samples, thereby also having an effect on the basicity. In this case, green solvents such as alcohols, ethanol, and methanol and water were investigated, resulting in the best catalytic performance for the Knoevenagel condensation between different substituted benzaldehydes and ethylcyanoacetates (the catalytic activity for the condensation between benzaldehyde and ethyl cyanoacetate is around 4.6 mol product h^−1^ g^−1^ catalyst at 80 °C in alcohols with total selectivity). 

In other words, the ammoxidation of commercial carbons—including carbon black and activated carbon (AC)—with ammonia and air was the strategy followed by Kan-nari et al. [131] to prepare *N*-doped carbons active for Knoevenagel condensation between ethyl cyanoacetate and benzaldehyde in 1-butanol at 90 °C. The catalysts derived from AC exhibited high activity levels as compared with the carbon black samples, while the porosity and surface area did not contribute to the observed reactivity differences. The authors highlighted that the nitrogen and oxygen functions on the carbon surface could act in cooperation in such a manner that the N/O ratio of approximately 1 resulted in the best catalytic activities (conversion = 96.9%; selectivity = 94.7%; yield = 91.8%). Similarly, Fujita et al. [132] developed a series of *N*-doped carbon materials via the ammoxidation of porous carbons prepared from commercial polyacrylonitrile. The catalytic performance during Knoevenagel condensation at 80 °C in 1-butanol, using benzaldehyde and ethyl cyanoacetate, of these materials depended on both the calcination and ammoxidation temperatures. The authors concluded that the ammoxidation produced basic sites that differed in nature from those generated by calcination. The presence of pyridine-type nitrogen functions modifies the electronic conjugated structure of the graphene surface, giving Lewis basic properties to the neighboring carbon atoms, which could be behind the enhancement of the catalytic activity (199 mmol g^−1^ h^−1^).

Much more recently, Li et al. [133] reported for the first time a very easy methodology for synthesizing novel series of basic *N*-doped porous carbons (Cz-MOF-253) from MOF-253, a MOF constituted by 2,20-bipyridine-5,50-dicarboxylic acid ligands coordinating Al^3+^. MOF-253 was directly pyrolyzed under an argon atmosphere at different temperatures—700, 800, 900, and 1000 °C—with subsequent Al^3+^ removal by treatment with HF (20%). The Cz-MOF-253 porous carbons showed medium-to-strong basic sites, with the main nitrogen species for all investigated samples being pyrrolic and pyridinic units, whereas graphitic N and oxidized N were present with lower contents. All *N*-doped porous carbons were active in the Knoevenagel condensation between benzaldehyde and malononitrile in toluene at 80 °C, although the Cz-MOF-253-800 sample exhibited the best catalytic activity, which was attributed to it having the largest surface area (conversion rates of up to 90% after 20 min). Different *N*-doped porous carbons were also synthetized from other MOFs such as ZIF-8 and Al-MIL-101-NH_2_ under identical conditions for comparison purposes, demonstrating the superior catalytic performance of the Cz-MOF-253-800 sample. This catalyst was also active when using differently substituted benzaldehydes. 

**Figure 5:**
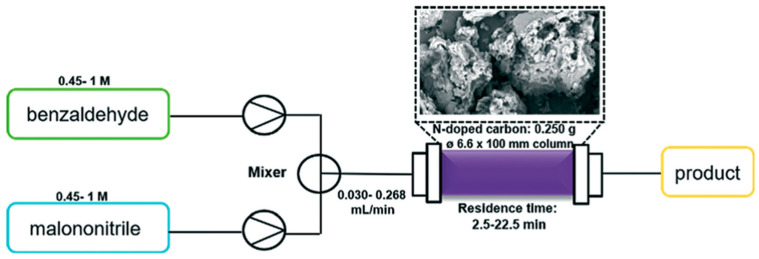
Flow system used in the Knoevenagel condensation between benzaldehyde and malononitrile in the presence of Cat-500. Copyright © 2021, Royal Society of Chemistry [134].

Recently, Brzęczek-Szafran et al. [134] developed a new family of mesoporous *N*,*O*-doped porous carbons prepared from glucose and the ionic liquid [EMIM][NCN] as carbon and nitrogen sources, respectively, as well as silica nanoparticles acting as a hard template, carbonized at different temperatures under an inert atmosphere. The catalysts were assayed during the Knoevenagel condensation between benzaldehyde and malononitrile in ethanol at 50 °C, yielding the desired product with high conversion and selectivity rates in the presence of Cat-500, the catalyst carbonized at 500 °C. Coinciding with the other previous reports mentioned above, pyridinic and pyrrolic species could be behind the observed catalytic performance. Interestingly, this condensation was investigated under continuous flow conditions using the Cat-500 catalyst, and the results were compared to those obtained for batch experiments (Figure 5). It was possible to sustainably prepare the desired product under mild reaction conditions, with excellent conversion rates (up to 95%) and the total selectivity reducing the unit operations, while also investigating the scope of the reaction by using different substituted benzaldehydes and even an aliphatic aldehyde such as heptaldehyde.

***Transesterification reactions*:** The activity of *N*-doped carbons prepared by Kan-nari et al. [131], firstly tested on Knoevenagel condensation, was also checked during the transesterification reaction between methyl acetate and ethanol. The maximum activity rates for both transformations were found when catalyzed by materials containing N/O ratios in the range of 0.8–1.0, suggesting that both nitrogen and oxygen functions on the surface act in cooperation. The authors proposed a cooperation model based on the pyridone function in which the activation of the acceptor (–CHO in benzaldehyde or –CO in methyl acetate) could be caused by hydrogen bonding with the –OH group in pyridine, with basic nitrogen sites activating the nucleophile (ethyl cyanoacetate or methanol), thereby explaining the N/O ratio of around 1. 

#### 3.1.2. Oxidation–Reduction Reactions

In recent years, metal-free *N*-doped porous carbons have been reported for oxidation–reduction reactions as promising candidates, possibly replacing in the near future the traditionally used metal- and metal-oxide-based catalysts. 

***Oxidation reactions*:** Several *N*-doped carbon materials have been reported for different oxidation processes, including alcohols and hydrocarbons, as well as for the oxidative coupling of amines. Watanabe et al. [135] reported on *N*-doped carbons prepared via the treatment of activated carbon with H_2_O_2_ at 100 or 130 °C, which were subsequently treated in a stream of ammonia or air at different temperatures—400, 600, 700, or 800 °C. These catalysts were applied in the aerobic oxidation of alcohols such as benzyl alcohol, cinnamyl alcohol, and 5-(hydroxymethyl)-2-furaldehyde, giving rise to corresponding aldehydes with low conversion rates but excellent selectivity (up to 93%). The authors concluded that the graphite-type nitrogen species could be considered the catalytic species, whereas the contribution of the oxygenated species to the catalytic performance was not found.

A family of *N*-doped carbons, synthetized from commercially available AC via thermal treatment in the presence of nitrogen sources such as pure ammonia, a mixture of ammonia and air, and NO applied in the aerobic oxidation of xanthene to xanthone was reported, although showing lower catalytic activity than commercial Ru/AC but higher than the traditional Pd/AC catalyst (yield = 38% vs. 25% for Pd/AC or 98% for Ru/AC) [136]. The use of a mixture of ammonia and air was the most efficient methodology to introduce nitrogen species over the carbon surface, such as pyridine, pyrrole or pyridone. In this case, a correlation between the catalytic activity and pyridine-type N/ether-type O species ratio was found, suggesting that the active sites were composed of inserted N atoms in a conjugated graphitic structure.

**Scheme 1:**
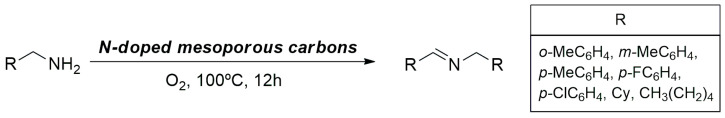
Oxidative coupling of methyl amines to the corresponding imines catalyzed by *N*-doped mesoporous carbon materials.

In continuation with the oxidation reaction of aromatic alkanes, *N*-doped carbons synthetized from MOFs have been reported. Yang et al. [137] developed a series of N, P, and S co-doped hollow carbon shells synthetized from ZnCo-ZIFs@PZS composites, where PZS is poly(cyclotriphosphazene-co-4,40-sulfonyldiphenol), exhibiting good catalytic performance for the selective oxidation of ethyl benzene and related compounds to the corresponding acetophenone, using *tert-*butyl hydroperoxide (TBHP) in water at 80 °C; the acetophenone was obtained with conversion rates of up to 84% and selectivity rates in the range of 92 to 99%. The synthesis strategy consisted of coating bimetallic ZIF-67 with hexachlorocyclophosphazene and 4,40-sulfonyldiphenol solution and triethylamine in different ratios, calcined at 900 °C under an argon atmosphere and finally submitted to sulfuric acid and water treatments. In the same context, highly graphitized *N*-doped mesoporous carbons prepared from MOFs by calcination (ZIF-67, ZIF-8, and Co-MOF-71) followed by treatment with aqua regia were investigated in the aerobic oxidation of cyclohexane at 125 °C, yielding adipic acid (conversion range of 15–48% with approximately 60% selectivity) [138]. In this case, the porosity was caused by the removal of metal nanoparticles via acid etching but also by adjusting the carbonization temperature. The selective oxidation of toluene to benzaldehyde was also investigated, although with lower conversion rates than those obtained in the presence of the conventional Pd/C catalyst. Interestingly, these catalysts were also active in the oxidative coupling of different benzylamines to the corresponding *N*-benzylidene benzylamines (yield = 78–99%), including acyclic and cyclic aliphatic amines, in the presence of oxygen at 100 °C (Scheme 1). The observed reactivity was due to both the homogeneous nitrogen distribution and the presence of mesopores.

*N*-doped carbons obtained from biomass, in this case via chitosan pyrolysis using urea as an additional nitrogen source, have been applied to the synthesis of 2,5-diformylfuran via the aerobic oxidation of 5-hydroxymethylfurfural under mild reaction conditions [139]. The catalytic activity was dependent on the concentration and type of N sites but also on the surface area, with both increasing with the pyrolysis temperature (conversion = 25–56%; selectivity = up to 90%). When increasing the pyrolysis temperature until 950 °C, in the NC-950 sample, the carbon layers were destroyed, producing abundant pores, whereas the carbon and nitrogen were uniformly distributed, although the latter had a lower content. The authors highlighted that the oxidation of 5-hydroxymethylfurfural was promoted by nitric acid, with the molecular oxygen acting as a secondary oxidant allowing nitric acid recovery. 

**Figure 6:**
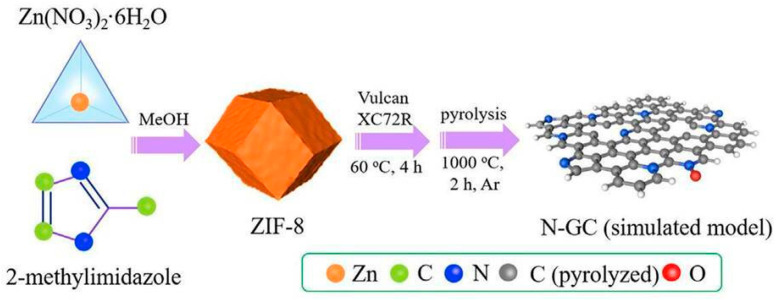
Synthesis and catalytic performance study of N-GC. Adapted from [140]. Copyright © 2018, Elsevier.

**Scheme 2:**
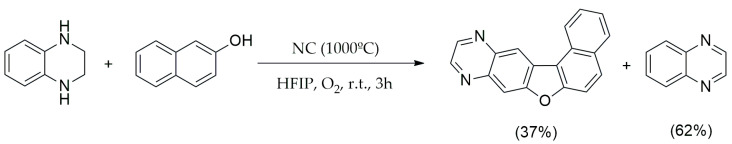
Reaction between tetrahydroquinoxalines and 2-naphthol at room temperature through oxidative aryl C-H bifunctionalization.

Much more recently, Zhao et al. [140] investigated a metal-free *N*-doped graphitic carbon catalyst, NC, from the direct co-carbonization of ZIF-8 and Vulcan XC72R in different ratios to other carbon sources at 1000 °C (Figure 6), whereby hexafluoroisopropanol (HFIP) was able to promote the reaction between a range of tetrahydroquinoxalines and 2-naphthols to π-extended quinoxaline(b)naphthofurans (isolated yields = 53–95%) at room temperature through oxidative aryl C-H bifunctionalization (Scheme 2). In this case, considering the elevated pyrolysis temperature, ZnO was sublimated, obtaining *N*-GC samples showing micro- (smaller than 2 nm), meso- (8 to 50 nm), and even macropores (larger than 50 nm). This hierarchically porous structure allows the interaction of molecular oxygen with active centers while the meso- and macroporous network favors better product diffusion. Note that a benzofuran-fused quinoxaline core is frequently contained in anticancer agents, organic light-emitting diodes (OLEDs), and photoelectronic materials.

**Scheme 3:**
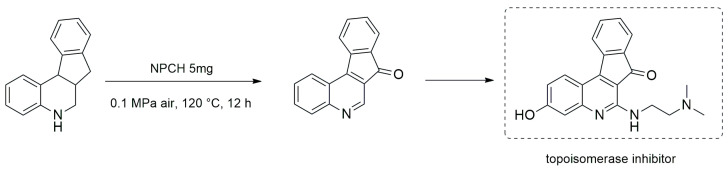
Synthesis of 7*H*-indeno(2,1-*c*)quinolin-7-one promoted by the NPCH catalyst in water with air.

In continuation with the synthesis of heterocycles, Sun et al. [141] very recently reported on the aerobic oxidative dehydrogenation of *N*-heterocycles under ambient conditions in water, catalyzed by *N*,*P* co-doped porous carbon as an attractive metal-free catalyst; it was prepared via the pyrolysis at 900 °C of ZIF-8 modified with benzylamine and different amounts of PPh_3_. In this case, the Zn phase was evaporated at the pyrolysis temperature while traces were removed by etching with HCl. The porous carbon NPCH was active in the oxidation of structurally different hydro-*N*-heterocycles, including tetrahydroquinolines, indolines, hydroquinoxalines, 1,2,3,4-tetrahydrobenzo(h)quinoline, and 1,2,3,4-tetrahydro-1,10-phenanthroline, among others, with some of the corresponding oxidated heterocycles even obtained on a multi-gram scale using low catalyst content. The authors applied the developed methodology to the synthesis of pharmaceutically relevant scaffolds such as 7*H*-indeno(2,1-*c*)quinolin-7-one as a precursor of the topoisomerase inhibitor TAS-102. In this case, both the dehydrogenation of hydroquinoline and benzyl oxidation took place in only one step (Scheme 3).

**Figure 7:**
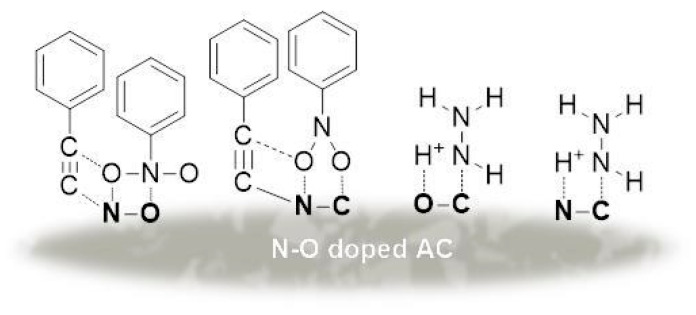
Interactions of nitrobenzene-*N*,*O*-doped AC with phenylacetylene.

***Reduction reactions*:** Fujita et al. [142] followed a similar strategy to prepare *N*,*O*-doped AC active for the transfer reduction of nitrobenzene, styrene, and 3-nitrostyrene in the presence of hydrazine hydrate as a hydrogen donor. The parent AC was submitted to different treatments with hydrazine, hydrogen peroxide, ammonia stream (90%), and air (10%) and heated at different temperatures of 400, 600, and 800 °C, also combining firstly the hydrogen peroxide treatment and subsequently the ammonia stream (90%) at 600 °C. All samples were moderately active in the reduction of nitrobenzene and nitrostyrene, the polar nitro group interacting with both oxygenated and nitrogenated species on the carbon surface. *N-* and *O*-dopant species should favor the formation of diimide and protons from hydrazine as reducing agents. Notably decreased activity was observed in the reduction of styrene, attributed to poor adsorption on the active species. Similarly, some of these authors investigated the reduction of phenyl acetylene [143], observing that it is possible when using mixtures in which phenylacetylene co-exists with nitrobenzene (conversion = 13–15%; selectivity to styrene = 73–78%). A plausible mechanism was suggested for the reaction, consisting of the adsorption of nitrobenzene through its nitro group over the *N*,*O*-doped carbon surface, allowing the adsorption of phenylacetylene (Figure 7).

Interestingly, *N*-doped porous carbon derived from biomass, particularly from radishes, has been used in 4-nitrophenol reduction in the presence of NaBH_4_, and also in styrene oxidation using *tert*-butyl hydroperoxide [144]. The catalyst was prepared in one pot by mixing the carbon precursor, urea, and KOH followed by carbonization under a N_2_ atmosphere, resulting in a *N*-doped carbon with hierarchical porosity and a large surface area (919–3063 m^2^ g^−1^). As mentioned above, the catalytic performance is more influenced by the graphitic N species content than the total N loading.

In the same context, nitrophenol reduction was also reported in the presence of *B–N* co-doped porous carbon materials (BNPCs) and NaBH_4_ at room temperature [145]. The BNPC was synthetized via the pyrolysis of adenine-based MOF-1 under a nitrogen atmosphere at 1000 °C, subsequently being treatment with boric acid and finally being submitted to thermal treatment at different temperatures. BNPCs are mesoporous samples presenting high degrees of graphitization, with both N and B being uniformly distributed. In this case, a synergy between dopants in the porous carbon materials, N and B, was proposed to explain the catalytic performance. In this way, Wang et al. [146] recently developed a biomass-derived *N*-doped porous carbon (NDC) active in 2-methyl-4-nitrophenol reduction in the presence of NaBH_4_. The authors stated that the *N*-doping in the carbon matrix is essential in this process, where the nitro group is preferentially adsorbed as mentioned above. NDC was prepared following a one-pot approach from a mixture of a dried powder of pumpkin, urea, and K_2_CO_3_ calcined at 800 °C under a nitrogen atmosphere and finally sequentially washed with hydrochloric acid solution, water, and ethanol. The NDC sample is an amorphous sample with highly developed microporosity, also containing mesopores and abundant defects and showing the uniform distribution of C, N, and O elements. The NDC presented a higher N loading on the surface (16.0 at. %), which was notably much more than the undoped sample (PC 0.58 at. %).

***Miscellaneous*:** Acetylene hydrochlorination is an important industrial technology for the production of polyvinyl chloride as a thermoplastic material. Lin et al. [147] developed a series of polyaniline-derived *N*-doped carbons, starting from polyanilines with different molecular weights, for this transformation as an interesting alternative to gold-based catalytic systems. The authors concluded that the high content of nitrogen, particularly pyrrolic functions, favors the reagents’ adsorption, whereas the good electrical conductivity probably has a positive effect on the diffusion of adsorbed species. 

### 3.2. Metal-Supported N-Doped Porous Carbon Catalysts

Metal-supported *N*-doped porous carbon catalysts have been explored in several interesting organic transformations. However, such materials have a new horizon ahead, especially regarding their application in fine chemical synthesis. In the subsequent sections, some interesting examples concerning the synthesis of valuable compounds are presented and discussed.

#### 3.2.1. Metal-Supported *N*-Doped Porous from Different Carbon and Nitrogen Sources

##### Oxidation Reactions

**Scheme 4:**
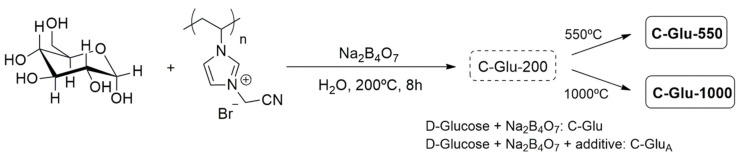
Strategy to synthesize *N*-porous carbons.

***Hydrocarbons and alcohol oxidation*:** Oxidation reactions including the oxidation of hydrocarbons and alcohols are the most investigated transformations promoted by metal-supported *N*-doped porous carbon obtained by using different synthesis approaches and varying both the carbon and nitrogen sources. In this regard, Zhang et al. [148] reported on one of the most popular catalysts, comprising TM nanoparticles supported on *N*-doped porous carbons (TM = Pd), which is highly active for the controlled oxidation of hydrocarbons and alcohols. Supports were prepared using the hydrothermal method from glucose and (poly)ionic liquids as abundant and cheap carbon sources and nitrogen-containing additives, respectively, at 550 and 1000 °C (Scheme 4). The C-Glu_A_-550 support was selected by the authors to prepare the corresponding Pd-supported catalysts due to its textural characteristics and *N*-doping; the ultrasonic-assisted deposition method was applied to obtain catalysts with different Pd loading rates (0.5–4 wt%). The catalysts were highly active for the solvent-free oxidation of ethyl benzene (TOF = 690 h^−1^; selectivity to acetophenone = 95%), benzyl alcohols, and others under atmospheric air or molecular oxygen conditions, giving rise to turnover frequencies that were notably superior to other Pd catalysts such as Pd@C, Pd@MgO, and Pd@TiO_2_, among others. The authors highlighted that the basic centers in Pd@C-GluA-550 could promote the O-H scission in the alcohol oxidation, while the heterojunction N-Pd enhances the metal’s electron density, favoring the oxidative addition of both O-H and C-H bonds to the Pd(0) nanoparticles.

In other words, Gil et al. [149] reported on different *N*-doped carbon nanospheres via the thermal pyrolysis of benzene (CNS_B_), aniline (CNS_AN_), or nitrobenzene (CNS_NB_) that were able to support gold nanoparticles and were applied for glycerol oxidation. He dispersion of the metallic phase influenced the glycerol conversion and glyceric acid selectivity, with both increasing when the gold particle size was decreased. Interestingly, while the Au/CNS_B_ induced the oxidation of primary alcohol groups in glycerol to glyceric acid (68% of selectivity), the *N*-doped catalysts, Au/CNS_AN_ and Au/CNS_NB_, selectively allowed the oxidation of the secondary hydroxyl group in glycerol. 

**Figure 8:**
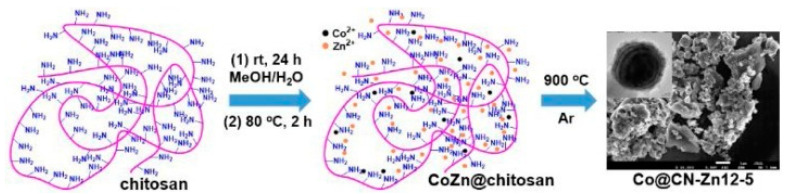
Synthesis of the Co@CN-Zn12-5 catalyst. Copyright © 2020, Elsevier [150].

***Oxidative esterification*:** Recently, Lin et al. [150] reported a hollow Co embedded in nitrogen-doped graphite (Co@CN) structure derived from biomass involved in the oxidative esterification of hydroxymethyl furfural to 2,5-furandicarboxylic acid dimethyl ester at 50 °C in the presence of O_2_ (1 atm). Chitosan and Zn(II) were used as sacrificial templates, proposing that both cations, Co(II) and Zn(II), could be coordinated to -NH_2_ or -OH functions in chitosan, affording CoZn@chitosan, which after pyrolysis (e.g., 900 °C) would generate a porous Co@CN sample when the partial evaporation of Zn was produced (Figure 8). The authors highlighted that zinc is the key in the synthesis of the materials and in the catalyzed reaction. The zinc acted as a sacrificial template but also the residual zinc in the porous sample could increase the Lewis basic sites, although while decreasing the strong Lewis acid sites activating the O-H and CH bonds at position α- in hydroxymethyl furfural and promoting the oxidation reaction.

##### Hydrogenation Reactions

Among the hydrogenation reactions catalyzed by metal-supported *N*-doped porous carbons are the hydrogenation of unsaturated compounds, olefins and alkynes, carbonyl compounds, and nitroarenes. The catalytic performance of various metal-supported (Pd, Rh, Ru, Ir) *N*-doped bamboo-like carbon nanotubes (BCNTs) compared to nitrogen-free multi-walled carbon nanotubes (MWCNTs) in octadecene hydrogenation has been reported [151]. Both catalyst types were prepared using the CVD method from *N*-butylamine and acetylene as nitrogen and carbon sources in the presence of Ni/MgO for BCNTs and Co-Fe/MgO catalysts in the case of MWCNTs. The metal-decorated carbon nanotubes were synthetized by impregnation with aqueous solutions of metal salts—PdCl_2_, [Rh(CH_3_CO_2_)_2_]_2_, (NH_4_)_2_IrCl_6_, and RuCl_3_—and calcinated under a nitrogen atmosphere at 400 °C. The catalytic performance of the Pd/BCNT was superior to that of the Pd/MWCNT sample, although with an increased Pd particle size, probably due to the formation of a stronger complex, as demonstrated by the theoretical calculations.

Much more recently, Liu et al. [152] easily developed new graphene-encapsulated Ni@N/C catalysts able to efficiently catalyze the semi-hydrogenation of alkynes to olefines with excellent activity (conversion = 43–99%) and selectivity (73–99%), in which the strong nitrogen and metal interactions were probably behind the observed selectivity. For the synthesis of Ni@N/C samples, different nitrogen sources such as urea, melamine, and ammonium citrate, among others, were used, influencing the final characteristics of the materials. 

Concerning the hydrogenation of carbonyl compounds, Ni(0)-supported *N*-doped AC (Ni/NAC) catalysts, prepared from AC and different amounts of melamine and pyrolyzed at three temperatures (700, 800, and 900 °C) under a nitrogen atmosphere, have also been reported for the hydrogenation of furfural in isopropanol under H_2_ pressure [153]. The investigated samples showed homogeneously dispersed Ni(0) particles, with both the pyrolysis temperature and melamine amount barely influencing the particle size. The Ni/NAC-1–1073 sample (starting from 0.5 g of AC and 1 g of melamine calcined at 900 °C) was found to be the most efficient catalyst, allowing tetrahydro-furfuryl alcohol to be selectively obtained with total conversion (3 h) at 80 °C; this reactivity was attributed to a synergistic effect of the nanosized Ni and nitrogen loading rates and types in combination with the large surface area and porous structure.

Interestingly, Lv et al. [154] reported on a new recyclable and magnetically separable catalyst consisting of γ-Fe_2_O_3_ nanoparticles modified with *N*-doped porous carbon (γ-Fe_2_O_3_/NPC) in the reduction of nitroarenes at 80 °C in ethanol, using hydrazine hydrate as the hydrogen donor (conversion = 47–99%; selectivity to aniline of up to 99%). The catalyst was prepared from a porous organic polymer, which was synthetized via the condensation between *p*-phenylenediamine and ferrocene carboxaldehyde, followed by pyrolysis under an inert atmosphere. As anticipated above, the γ-Fe_2_O_3_ nanoparticles in combination with the *N*-doped porous carbon allowed both the hydrazine’s adsorption and activation, producing active hydrogen atoms. 

**Figure 9:**
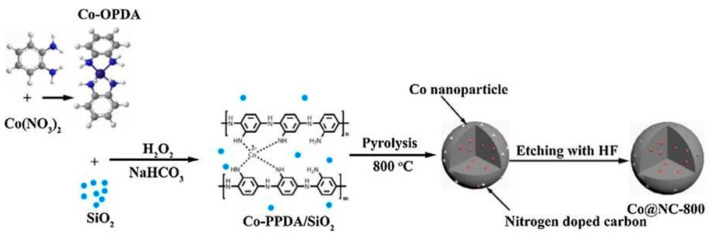
Synthesis of the Co@NC-T catalysts. Copyright © 2019, Elsevier [155].

In another context, a magnetically separable cobalt-supported *N*-doped carbon was developed for the selective reductive amination of carbonyl compounds to primary amines in the presence of H_2_ and ammonia [155]. The catalysts were obtained from Co(II) salt, *o*-phenylendiamine, and SiO_2_ as hard templates, as shown in Figure 9, and pyrolyzed at different temperatures. The Co@NC-600 sample prepared at 600 °C was highly active and selective in the reductive amination of benzaldehyde to *N*-benzylidenebenzylamine (yield = 90%); in the presence of catalysts obtained at superior pyrolysis temperatures (800 or 900 °C), the residual formation of benzylamine and dibenzyl amine as byproducts was observed.

##### Miscellaneous

**Scheme 5:**
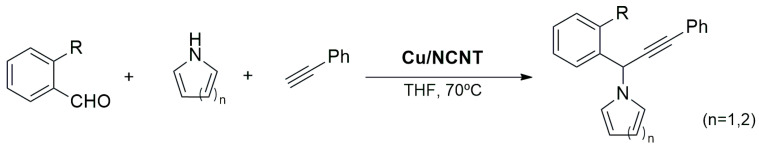
Synthesis of propargylamines catalyzed by Cu nanoparticles stabilized on *N*-doped carbon nanotubes.

***Coupling reaction to propargylamines*:** Ramu et al. [127] stabilized metal Cu nanoparticles on *N*-doped carbon nanotubes for the clean multicomponent synthesis of propargylamines (yield = 80–83%), as interesting intermediate species of pharmaceuticals, under mild reaction conditions (Scheme 5). The catalysts were prepared from oxidized CNTs treated with ammonia and heated at different temperatures ranging from 200 to 800 °C. Finally, the *N*-doped CNTs were impregnated with a copper acetate aqueous solution (10% wt) and subjected to an additional thermal treatment.

**Scheme 6:**
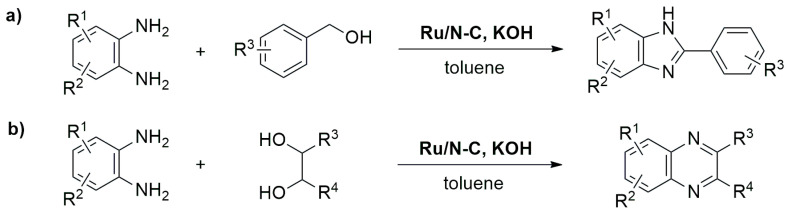
Cross-coupling reactions of diamines with (**a**) alcohols to benzimidazoles and (**b**) diols to give quinoxalines.

***Heterocyclic synthesis*:** Heterocycles are compounds of great importance because these compounds represent structural motifs found in a huge number of biologically relevant natural or synthetic drugs, agrochemicals, and medicines, among others. In this regard, Guo et al. [156] developed a new catalyst comprising Ru nanoparticles (Ru(0) and RuO_x_) supported on *N*-doped carbon (Ru/N@C) for the synthesis of *N-*containing heterocycles, such as benzimidazoles (yield = 73–90%) and quinoxalines (yield = 21–81%), obtained with good to excellent yields at 110–130 °C in toluene and in the presence of KOH, following a strategy known as acceptorless dehydrogenation coupling (ADC) (Scheme 6). The catalyst was prepared via pyrolysis at 800 °C under an inert atmosphere of *cis*-Ru(*phen*)_2_Cl_2_ supported on carbon (VULCAN XC72R). In addition, Ru/N@C samples with increased N contents were also synthetized by adding increased *phen* amounts to the mixture before carbonization, with the Ru particle size decreasing with the N loading. This methodology has been also explored in the reaction of amines with alcohols to imines and secondary amines, showing that the selectivity was controlled by the alkali metal composing the base (NaOH or KOH). The authors proposed that the reaction takes place as follows: (i) dehydrogenation of alcohol to the corresponding aldehyde; (ii) condensation with amine to give the corresponding imine; (iii) hydrogenation of the formed imine to the corresponding secondary amine. While the dehydrogenation of the starting alcohol was produced in the presence of any base (NaOH or KOH), the hydrogenation of imine depended on the used base. In the presence of NaOH, the imine was not hydrogenated, whereas when using KOH, the formation of the corresponding secondary amine was observed.

**Scheme 7:**
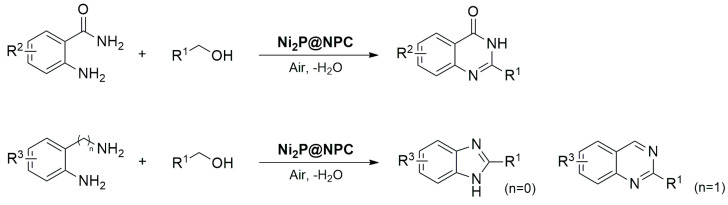
Synthesis of *N*-heterocycles promoted by the Ni_2_P@NPC-800 catalyst.

Much more recently, Song et al. [157] developed a new recyclable and inexpensive catalyst composed of highly stable ultrafine Ni_2_P nanoparticles dispersed on *N,P*-co-doped biomass-derived porous carbon (Ni_2_P@NPC-800) for the synthesis of heterocycles, such as quinazolines, quinazolinones, and imidazoles, as pharmaceutically relevant *N*-heterocycles. The reaction occurred via oxidative cross-dehydrogenative coupling between alcohols and diamines or 2-aminobenzamides under aerobic conditions in toluene at 120 °C, in the presence of *^t^*BuOK as the base (Scheme 7). Note that this methodology showed good tolerance to a variety of functional groups. The catalyst was synthetized from a biochar prepared from bamboo shoots and treated with an aqueous solution of Ni(OAc)_2_ and phytic acid as Ni and P precursors, which was finally pyrolyzed at 800 °C under a N_2_ atmosphere. The resulting sample presented hierarchical micro-, meso-, and macropores showing large surface areas and pore volumes.

#### 3.2.2. Metal-Supported *N*-Doped Porous Carbons from MOFs

As anticipated, the main MOF types used to prepare *N*-doped porous carbons are ZIFs, although they are not the only ones. Porous carbons from MOFs built from others nitrogen organic linkers than imidazole and derivatives, or even using additional nitrogen sources during the synthesis, have been also reported. It is also possible to prepare SACs starting from MOFs, and some of these examples used for fine chemical synthesis are mentioned below. These *N*-doped porous carbons have been widely applied in oxidation reactions, mainly in the oxidation of alcohols, hydrocarbons, and epoxidation reactions but also in reduction reactions principally involving the reduction of nitroaromatics and aldehydes and the hydrogenation of aromatics [46,158]. Some interesting examples of metal-supported *N*-doped porous carbons from MOFs that are useful in fine chemical synthesis are commented on in the subsequent sections.

##### Oxidation Reaction

**Scheme 8:**
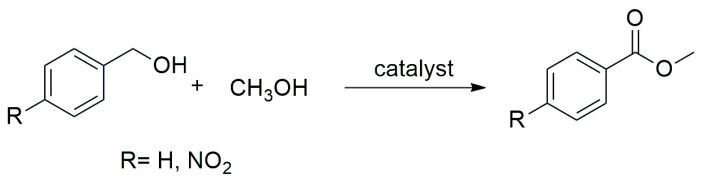
One-pot oxidative esterification of benzyl alcohols catalyzed by *N*-doped porous carbons from ZIF-67.

***Alcohols to esters*:** The one-pot synthesis of esters from alcohols consisting of two steps, alcohol oxidation and esterification, has been reported by using *N*-doped porous carbons containing Co nanoparticles prepared from MOFs, in particular ZIF-67 (Co(MeIm)_2_), an MOF with good thermal stability and high nitrogen and carbon contents [159,160]. Co@C-N composites in each case were prepared by the direct pyrolysis of ZIF-67 under an inert atmosphere (N_2_ or Ar) at different temperatures (500, 600, 700, 800 and 900 °C), resulting in microporous structures. Both studies described the esterification of benzyl alcohols by varying the reaction conditions (benzyl alcohol, O_2_ (1bar), K_2_CO_3_, 80 °C vs. *p*-nitrobenzyl alcohol air, 25 °C) in the absence of any base, giving the corresponding methyl ester with high selectivity (99%) (Scheme 8). These differences probably were due to the superior reactivity of *p*-nitrobenzyl alcohol but also to the presence of different Co phases. While the catalysts prepared by Zhou et al. [160] contained Co and CoO nanoparticles, Zhong et al. [159] reported the exclusive presence of metallic Co.

##### Reduction Reaction

The reduction of carbonyl compounds either by hydrogenation or hydrosilylation constitutes an important transformation in organic synthesis. Long et al. [161] developed a series of Co-based *N*-doped carbon catalysts of Co@C-N from Co-MOFs ([Co(bdc)(ted)_0.5_]·2 DMF·0.2 H_2_O) via direct pyrolysis at different temperatures, which were tested in transfer hydrogenation reactions of a plethora of different unsaturated compounds such as aryl and aliphatic ketones, nitriles, nitro compounds, and olefins at 80 °C in isopropanol. The catalyst pyrolyzed at 900 °C, Co@C-N-900-15h, showed the best catalytic performance, affording the desired product with high selectivity rates (up to 90%) and excellent conversion values (80–99%). This catalyst was also found to selectively catalyze the transfer hydrogenation reaction of nitriles to primary amines or even imines with controllable selectivity (up to 90%), depending on the used hydrogen donor (isopropanol in this case) volume, in such a way that the use of large amounts of isopropanol yielded the corresponding benzyl imine [8]. The observed reactivity was rationalized as (i) proton transfer from isopropanol to activate benzonitrile, (ii) with both adsorbed over the catalyst’s surface, affording acetone and the corresponding primary amine, (iii) followed by hydrogenation to benzyl amine, which (iv) reacted with another imine molecule, leading to an intermediate compound that underwent ammonia release, producing the corresponding imine. In the same context, transition metal alloy nanoparticles embedded in *N*-doped carbon derived from multimetallic M-M′-MOF, ([(M-M′(1,4-bdc)_2_(dabco)] · 4DMF · ½H_2_O, where M/M′ = Co, Ni, Cu) have been explored [162]. Co-Ni alloy nanoparticles encapsulated in *N*-doped graphitic carbons have been also investigated for the hydrosilylation of ketones exhibiting good catalytic performance [163]. The alloy CoNi materials were prepared from ZIF-67, a Co-MOF, by incipient wetness impregnation with nickel(II) nitrate and subsequently carbonized at different temperatures.

##### Miscellaneous

**Scheme 9:**
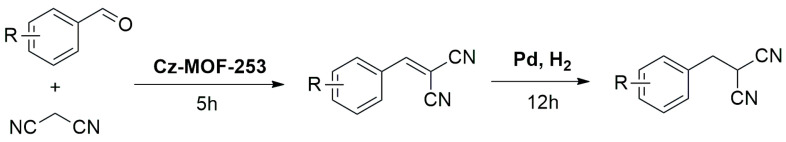
Knoevenagel condensation–hydrogenation reactions between substituted benzaldehydes and malononitrile promoted by the Pd/Cz-MOF-253-800 catalyst.

Although metal MOF-derived *N*-doped carbon catalysts have been mainly explored in oxidation–reduction reactions, there are at least two reported examples involved in acid–base reactions. Such is the case of the basic *N*-doped porous carbon, Cz-MOF-253, tested in the Knoevenagel condensation reaction between benzaldehyde and malononitrile [133]. Cz-MOF-253 comes from MOF-253, in which Al^3+^ is coordinated to the 2,20-bipyridine-5,50-dicarboxylic acid ligand. MOF-253 was carbonized at different temperatures and afterwards Al^3+^ was removed by treatment with HF. In the case of Cz-MOF-253-800, which was carbonized at 800 °C, the presence of medium to strong Lewis basic sites exhibiting excellent catalytic performance was observed. In addition, the authors interestingly reported on the catalyst Pd/Cz-MOF-253-800, in which Pd nanoparticles were supported, which is active in the tandem catalytic Knoevenagel condensation–hydrogenation reactions by reacting different benzaldehydes with malononitrile in toluene, as shown in Scheme 9.

**Scheme 10:**
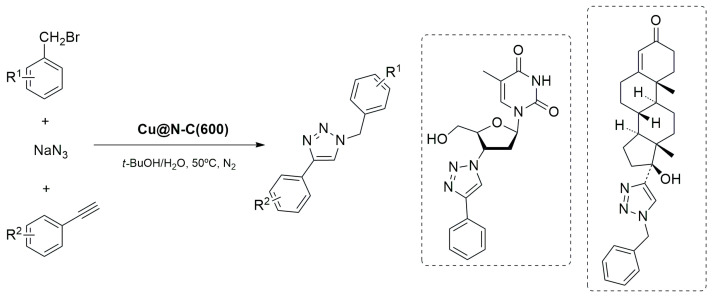
Synthesis of 1,4-disubstituted 1,2,3-triazoles from terminal alkynes, aryl halides, and sodium azide promoted by the Cu@N-C(600) catalyst.

Another recent example of the application of this type of material consisted of the development of MOF-derived catalysts for the synthesis of 1,4-disubstituted 1,2,3-triazoles in high yields (70–98%) by the 1,3-dipolar cycloaddition of terminal alkynes, aryl halides, and sodium azide, a relevant heterocyclic system in the pharmaceutical industry [164] (Scheme 10). The authors easily prepared a series of Cu@N-C catalysts from copper(II) bisimidazolate (Cu(Im)_2_) via direct pyrolysis by varying the temperature from 400 to 800 °C. The thermal treatment of the selected MOF provoked the modification of its structure, giving a *N*-doped porous carbon with a flat structure. The Cu(II) in the MOF was in situ reduced to Cu(0), which gradually tended to form aggregates when increasing the pyrolysis temperature, also increasing the Cu(0) content. All samples were active in the investigated reaction, with the Cu@N-C(600) catalyst showing the best catalytic performance, even being superior to the traditional catalytic system (CuSO_4_/sodium ascorbate). The developed methodology was investigated over a broad substrate range, tolerating a wide range of functional groups.

**Scheme 11:**
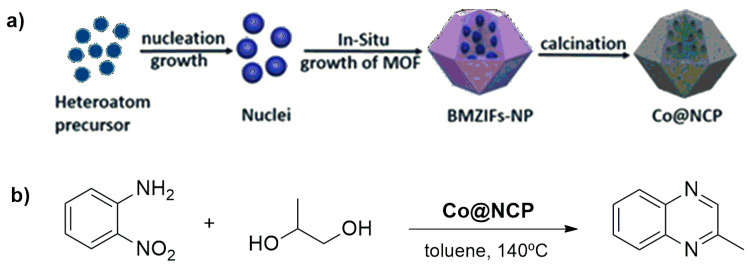
(**a**) Synthesis procedure used to obtain *N,P* Co-doped cobalt catalysts. Copyright © 2020, Wiley-VCHGmbH [156]. (**b**) Synthesis of quinoxaline.

A similar ADC synthesis approach for the synthesis of quinoxalines to that used by Guo et al. [156] has been reported by using a *N*,*P* Co-doped cobalt catalyst derived from an MOF [165]. The catalysts of Co@NCP were prepared from bimetallic zeolitic imidazolate frameworks (Co(II) and Zn(II)) following a sequence involving (i) heteroatom precursor nucleate (ammonium phosphate), (ii) in situ ZIF growth, and (iii) pyrolysis (Scheme 11). 

As already mentioned, the Zn was evaporated during the thermal treatment, resulting in a porous carbon with an increased surface area. In this case, the catalysts were highly efficient in the reaction between 1,2-propanediol and *o*-nitroaniline in toluene at 140 °C under base-free conditions, giving the corresponding quinoxalines in moderate to high yields (49–87%). The great catalytic performance was attributed to (i) the presence of small Co nanoparticles, which were highly dispersed over the carbon surface; (ii) the formation of Co-P bonds, optimizing the coordination environment of the cobalt sites, improving the hydrogen transfer; and (iii) N and P doping, which increased the Lewis basicity in the Co@NCP required for the dehydrogenation of glycol and cyclization. Note that this strategy has been also reported by Panja et al. [166] using Co-*phen*/C-800 carbon. The carbon catalysts were prepared from the cobalt precursor, phenanthroline, and carbon powder (carbon black). In this case, the reaction required higher temperatures (150 °C during 24 h) and the use of a base such as CsOH.

#### 3.2.3. Single-Atom Catalysts Derived from *N*-Doped Porous Carbons

SACs supported on different *N*-doped porous carbons have been developed and applied in several oxidation–reduction reactions. Such is the case for TM (TM = Fe or Ni) *N*-doped graphene catalyzing the oxidation of hydrocarbons (such as benzene or methane) [167,168] and selective CO_2_ reductions [169]. 

**Scheme 12:**
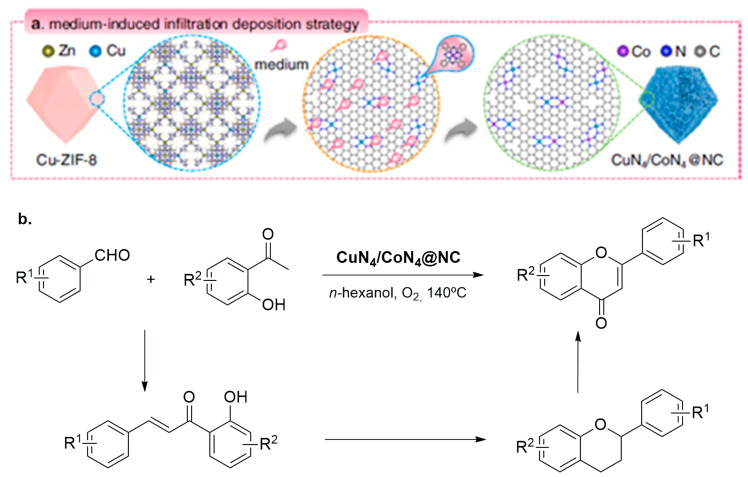
(**a**) Synthesis strategy used for CuN_4_/CoN_4_@NC [170]. (**b**) Synthesis of flavones catalyzed by hetero-SAC CuN_4_/CoN_4_@NC.

In continuation of the above section, some SACs have been properly prepared from MOFs. As a recent example, Zhao et al. [170] reported a new family of dual-metal hetero-SACs embedded on *N*-doped carbons (M_a_N_4_/M_b_N_4_@NC, M_a_ = Cu, Co, Ni, Mn; M_b_ = Co, Cu, Fe) exhibiting excellent catalytic activity in flavone synthesis (with yields ranging from 70 to 99%). In Scheme 12, the synthesis approach used to prepare the catalysts is summarized, which allowed the atomic dispersion at the same time as controlling the conversion of non-metallic species. M_a_-containing MOFs and M_b_-based phthalocyanine (Ph) were pyrolyzed in a mixed molten salt (KCl-KBr). During the process, Co-Ph infiltrated inside the MOF while decomposing to produce CuN_4_ and CoN_4_ sites embedded on the *N*-doped carbon matrix with a hierarchical porosity when the KCl-KBr was removed (partial Zn removal took place at high temperatures). The best catalytic performance observed for the CuN_4_/CoN_4_@NC catalyst may have been due to both metal sites, namely CoN_4_ and the vicinal CuN_4_, being homogeneously distributed, which act in cooperation, favoring O_2_ activation, as experimental and theoretically demonstrated, while interconnecting hierarchical pores facilitated the diffusion of both the reagents and products.

Wang et al. [171] also reported an interesting example consisting of single Ru atoms supported on *N*-porous carbons that were highly active dur quinoline hydrogenation in the presence of H_2_ at 100 °C, affording tetrahydroquinoline with both high conversion and selectivity rates (99%). The developed methodology allowed the authors to selectively prepare relevant biologically active heterocycles such as 1,2,3,4-tetrahydro-6- or 8-hydroxyquinolines. The catalyst was prepared from UiO-66, Zr_6_O_4_(OH)_4_(BDC)_6_ (BDC = 1,4-benzenedicarboxylate), and UiO-66@NH_2_ by adding RuCl_3_ during the synthesis, with Ru(III) interacting with the amine functions. After the pyrolysis at 700 °C followed by the ZrO_2_ crystals’ removal via treatment with HF solution, the Ru SAs/N@C carbon with both a large pore volume and surface area was obtained. Ru(III) species comprising Ru single atoms or small Ru clusters were detected in the Ru SAs/N@C sample, whereas Ru clusters were predominant in the final porous carbon without amine functions.

## 4. Concluding Remarks

Carbon materials acting as either supports or catalysts have been applied in heterogeneous catalysis for a long time because of their fascinating properties, emphasizing their large surface areas and tunable chemical surface and porosity levels. In the last decades, the knowledge concerning *N*-doped porous carbons has led to great progress in such a manner that *N*-doping or even doping with other foreign species is considered as an appropriate strategy to modify the carbon properties adapted for a specific use. In this regard, different strategies and methodologies for the synthesis of these materials from diverse precursors, including biomass, different carbon and nitrogen sources, or even MOFs and COFs, have been reported. The selection of the precursor and synthesis method will depend on the desired properties and the final application.

*N*-doped carbon materials can be considered as a new class of metal-free catalysts, although they are notably less explored than the corresponding undoped ones for several organic transformations involving base-catalyzed reactions such as Knoevenagel condensation and esterification, and also in oxidation–reduction reactions. Especially interesting is the case of *N*,*O*-doped carbons implied in the reduction of nitrobenzene, where the polar nitro group probably interacts with both *O*- and *N*-doped species, also favoring the formation of active species from hydrazine [142,143]. In addition, *N*-doped carbon materials could represent sustainable and promising alternatives for the development of basic catalysts with great potential for fine chemical synthesis.

Nevertheless, *N*-doped carbon-supported metal catalysts often exhibit superior catalytic performance in a variety of industrially relevant catalytic reactions, probably attributed to the stronger interaction between the nitrogen and metallic phase greatly favoring a high nanoparticle dispersion. In fact, these materials represent an excellent opportunity to design SACs that are active in the synthesis of valuable compounds, a current research field that is still to be developed. 

The rational design of catalysts based on *N*-doped carbon with applications in diverse fields will be possible sooner rather than later. For this, extensive experimental studies concerning the synthesis and characterization of these materials in conjunction with structure–activity relationships and even theoretical studies for the identification of the most probable catalytic sites by studying the possible coordination environments will be the guide to designing the optimal catalysts for the synthesis of valuable compounds, particularly for the synthesis of heterocycles through cascade reactions, which is still in its infancy. In addition, the use of machine learning techniques in catalysis as powerful tools for the interpretation of large catalyst datasets will contribute in the near future to this progress [172]. 

However, despite their intensive study and the general idea among researchers of the immense potential of these materials, there are some limitations for *N*-doped carbon catalysts and their industrial applicability. Even if some authors consider there no need to further invest in the development of new doped carbon materials, since the advantages of doping are already established, suggesting efforts should be diverted elsewhere [173], a deeper understanding of the role of the different dopants over the material properties is necessary. Some synthesis procedures are complex and involve multiple steps, hindering their scalability for industrial production, which may also cause reproducibility difficulties. On the other hand, Kicinski and Dyjak [174] presented a careful discussion of the influence of impurities in carbon materials, particularly transition metal impurities, which can be very relevant in catalytic applications. All of this suggests that this topic will remain an active field of research, with increased efforts made to reach commercial readiness. 

## Data Availability

Not applicable.
